# Ureteral tumor with morphological features analogous to phyllodes tumor: a unique case with concomitant urothelial carcinoma

**DOI:** 10.1186/s13000-022-01277-6

**Published:** 2022-12-23

**Authors:** Wei Zhou, Linlin Dang, Chuangang Chong, Xiankui Cheng, Yejun Qin, Wenjing Su

**Affiliations:** 1 Department of Stomatology, Shandong Provincial Hospital Affiliated to Shandong First Medical University, Jinan, China; 2 Department of Pathology, Shandong Cancer Hospital and Institute, Shandong First Medical University, Shandong Academy of Medical Sciences, Jinan, China; 3 Department of Pathology, The Second People’s Hospital of Heze, Heze, China; 4 Department of Pathology, Shandong Provincial Hospital Affiliated to Shandong First Medical University, 324 Jingwuweiqi Road, Huaiyin District, 250021 Jinan, Shandong China

**Keywords:** Bladder, Phyllodes tumor, Ureter, Urothelial carcinoma, Case report

## Abstract

**Background:**

Phyllodes tumors belong to a spectrum of biphasic fibroepithelial lesions and are most commonly found in the breast. They are extremely rare in the urinary tract and only one case of bladder phyllodes tumor has been reported.

**Case presentation:**

We present a 69-year-old man with gross hematuria without an apparent cause. Computed tomography-urography and cystoscopic examination revealed a 5 × 4 cm lesion in the right ureteral orifice. He underwent a laparoscopic nephroureterectomy and partial cystectomy. Postoperative pathology confirmed a leaf-like structure consisting of myxoid stroma and peripheral urothelium. Stromal cells were spindle-shaped and stellate in appearance with no conspicuous cytological atypia or mitosis. The outlining urothelium had varying degrees of dysplasia, while in areas with moderate-to-severe dysplasia, active mitotic activity, abnormal giant cells, and focal early infiltration were observed. Overall, this case had the morphological features of benign phyllodes tumors and concomitant invasive urothelial carcinoma inside. The patient remained disease-free at 7 months after surgery.

**Conclusion:**

We report the first ureteral tumor with the morphological characteristics of a phyllodes tumor and concomitant invasive urothelial carcinoma inside. Considering the potential for local recurrence of phyllodes tumors and invasive urothelial carcinoma, long-term clinical and radiological follow-up of such lesions are advisable.

## Background

Phyllodes tumors (PTs) belong to a spectrum of fibroepithelial lesions with epithelial–stromal biphasic differentiation and are most common in the female breast. In the breast, combined assessment of a series of morphological features, such as stromal cellularity, cell pleomorphism, mitotic activity, and the presence of infiltrative growth at the tumor border, allows classification of PTs into benign, borderline, and malignant.

PTs in the male urogenital tract are rare. Less than 100 cases of PT in the prostate [1, 2], fewer than 15 cases in seminal vesicles [2, 3], and rare cases in the verumontanum [2] have been described to date. Only one case of bladder PT has been reported in the human urinary tract, which was a low-grade PT in a 54-year-old male [4]. There is also a report of bladder PT in a female rat [5]. PT in the ureter has not been reported previously.

Here, we report a case of a 69-year-old man who presented with a unique ureteral tumor with the morphological features of a breast PT. To our knowledge, this is the first such case described in the ureter.

## Case presentation

**Fig. 1:**
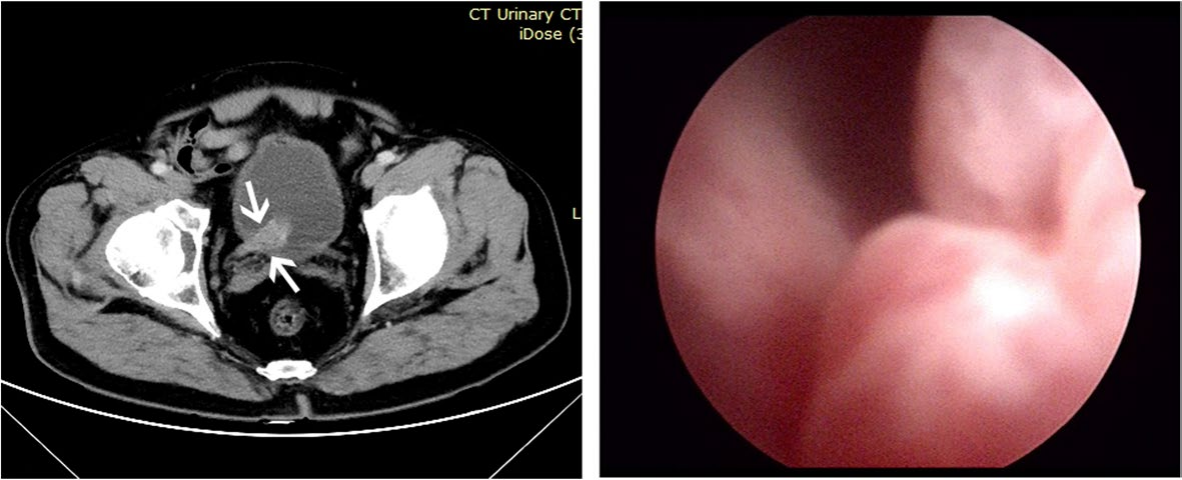
Representative views of the ureteral tumor by computed tomography-urography (CTU) and cystoscopy. **a** CTU showed an enlarged soft tissue shadow along the pelvic segment of the right ureter and continuous bladder trigone. **b** Cystoscopic examination revealed an irregular, polypoid, and pedicled lesion in the ureteral orifice

A 69-year-old Chinese man with a history of myocardial and cerebral infarctions presented with gross hematuria without an apparent cause. He did not experience frequent urination, urgent urination, dysuria, fever, dizziness, or flatulence. He was treated with antibiotics, but the effect was unsatisfactory. Computed tomography-urography (CTU) revealed an enlarged soft tissue shadow along the pelvic segment of the right ureter and the continuous bladder trigone (Fig. 1a), which was suspected to be urothelial carcinoma by radiologists. CTU also revealed abnormalities such as dilatation and ureteral stones in the right upper ureter, a shrunken and hydronephrotic right kidney, bilateral renal cysts, and prostatic hyperplasia. Cystoscopic examination revealed an irregular, polypoid, lobulated, and pedicled lesion measuring 5 × 4 cm in the right ureteral orifice (Fig. 1b). The right ureteral orifice was not visible. There was no other noticeable abnormality in the rest of the bladder mucosa. Biopsies were obtained from the mass for histological diagnosis. Then, he underwent a laparoscopic nephroureterectomy and partial cystectomy without subsequent radiation or chemotherapy. The patient remained disease-free at 7 months after surgery.

**Fig. 2:**
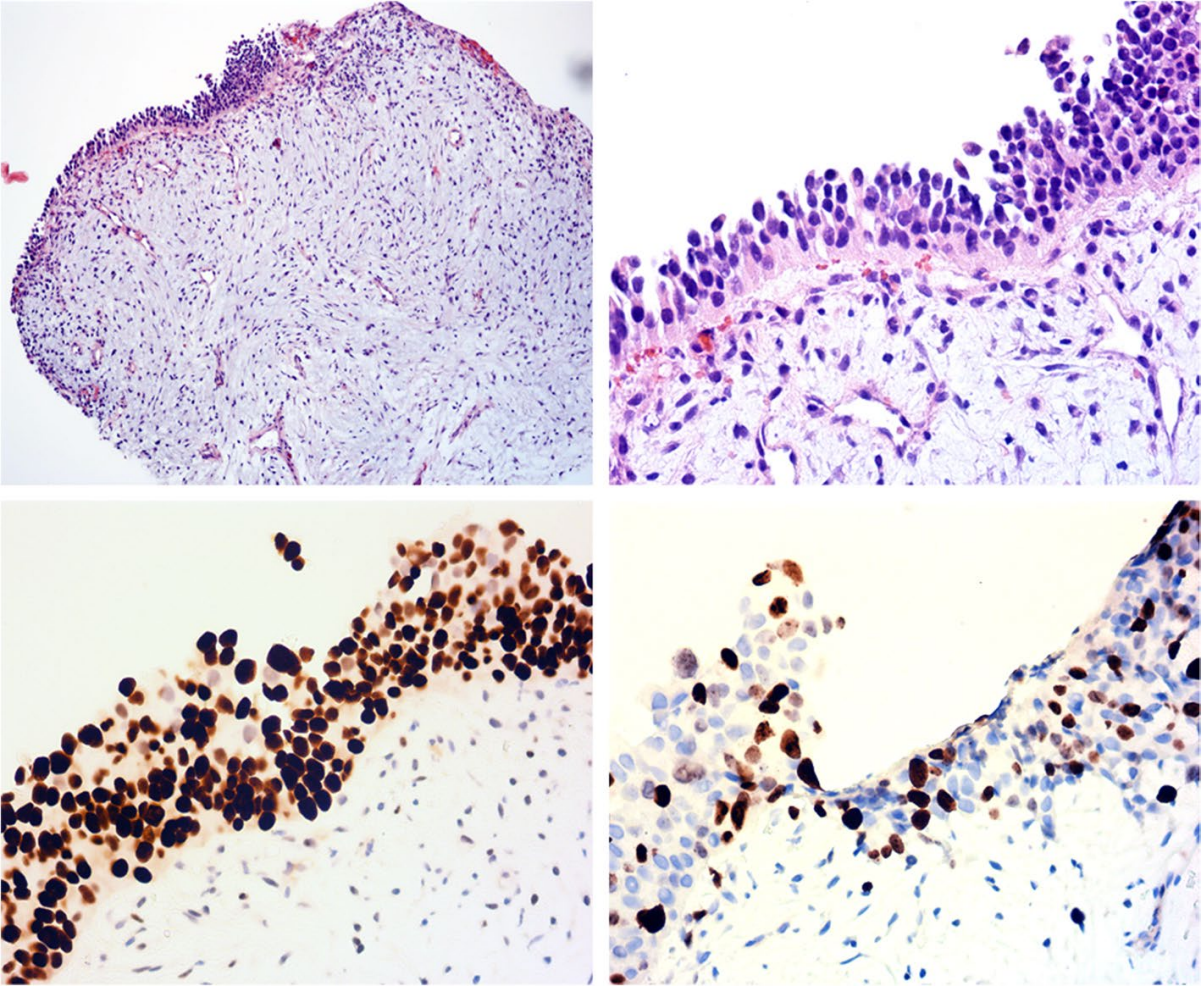
Histological findings of the biopsy sample. **a** Low power view showed a stromal–epithelial lesion (×40). **b** The urothelial epithelium lining the surface showed mild-to-moderate dysplasia, cell polarity disorder, and inconspicuous mitosis. The myxoid stroma showed no dense cellularity, significant atypia, or mitosis (×400). A diffuse expression pattern of p53 (**c**) and a relatively high proliferation index determined by Ki67 staining (**d**) were found in the urothelium (×400)

A biopsy sample from the lesion showed a stromal–epithelial lesion (Fig. 2a). The urothelial epithelium lining the surface showed mild-to-moderate dysplasia, cell polarity disorder, and inconspicuous mitosis (Fig. 2b). These histological changes did not justify a diagnosis of urothelial carcinoma in situ, although the epithelium immunostained diffusely for p53 (Fig. 2c). The proliferation index of the epithelia was approximately 15% as determined by Ki67 staining in hot spots (Fig. 2d). We also initially observed an abnormal mucoid change of the stroma. However, no dense cellularity, significant atypia, or mitosis was detected there with a relatively low proliferation index (Fig. 2d, lower portion). CD34, CD99, and Bcl2 were all negative in stromal cells. Finally, a diagnosis of dysplasia of urothelial epithelium with myxoid stroma was made.

Gross examination revealed a unilateral kidney, a ureter connected to the kidney, and two tumor fragments. The sizes of the tumor fragments were approximately 4 × 2.5 × 2 cm and 1.5 × 1 × 0.5 cm. The fragments were multi-nodulated with a solid, gray, and translucent cut surface. No bleeding or necrosis was noted in the lesion. Ureterectasis, ureteral stones, and right kidney atrophy were also noted.

**Fig. 3:**
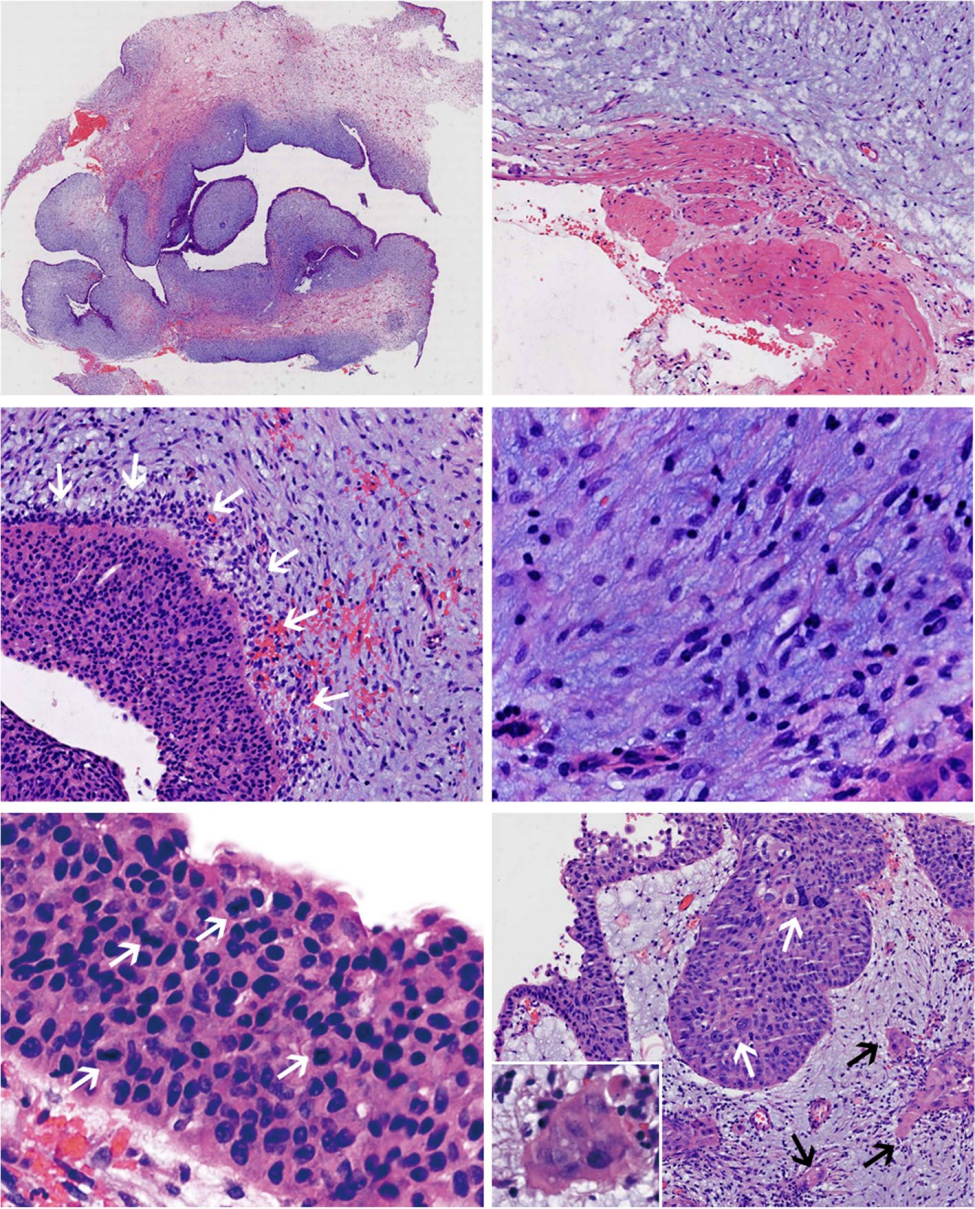
Histological findings of the resected tumor. **a** Scanning view of a hematoxylin and eosin-stained section showed a polypoid and lobulated tumor arising from the ureteral mucosa. **b** A relatively clear border was observed in the periphery of the lesion (×100). **c** Subepithelial condensation of stromal cells was noted (×200). **d** Stromal cells were spindle-shaped and stellate in appearance, and no conspicuous cytological atypia or mitoses were found in the stroma (×400). **e** Active mitosis was detected in areas with moderate dysplasia (×400). **f** Some abnormal giant cells (white arrows) and early infiltration (black arrows) were detected in areas with severe dysplasia (×400). At high magnification, the microinvasive carcinoma component was composed of irregular nests of oval epithelial cells with abundant eosinophilic cytoplasm (f, left lower)

Microscopic examination revealed a polypoid stromal–epithelial tumor arising from the mucosa. The tumor was arranged in a leaf-like and clefting pattern (Fig. 3a), which was reminiscent of a breast PT. There was no envelope around the tumor, but a relatively clear boundary was observed between the mass and the muscularis propria of the ureter (Fig. 3b).

The leaf-like structures consisted mainly of myxoid stroma, the periphery of which was lined by urothelium. The blue-stained stromal component was myxoid, and subepithelial condensation of stromal cells was noted (Fig. 3c). Stromal cells were spindle-shaped and stellate in appearance. No conspicuous cytological atypia or mitoses were found in the stroma (Fig. 3d).

Concerning the urothelial epithelium lining the stoma, varying degrees of dysplasia were observed. In areas with moderate-to-severe dysplasia, active mitotic activity (Fig. 3e), abnormal giant cells (Fig. 3f, white arrow), and early infiltration (Fig. 3f, black arrow) were noted. At high magnification, the invasive carcinoma consisted of irregular nests of oval epithelial cells with abundant eosinophilic cytoplasm (Fig. 3f, lower left).

**Fig. 4:**
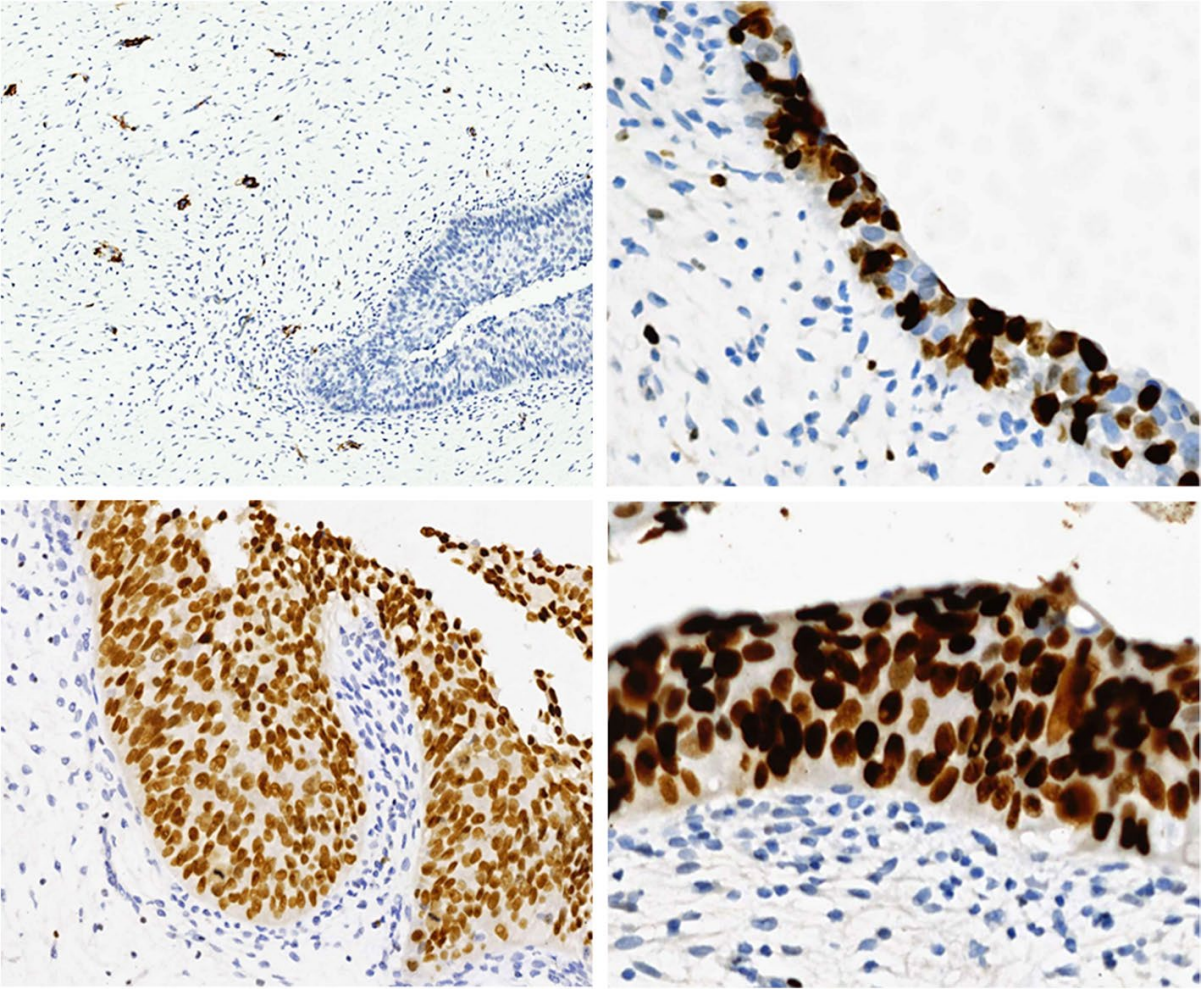
Immunohistochemical findings of the resected tumor. **a** Negative staining of SMA was observed in the stromal component (×100). **b** The proliferation indexes of urothelial and stromal cells were 60% and 3%, respectively, as determined by Ki-67 staining (×200). The urothelial epithelium was diffusely positive for GATA-3 (**c**) and p53 (**d**) (×400)

Immunohistochemistry showed that the stromal cells were negative for a panel of markers, such as SMA (Fig. 4a), CD117, CD34, and Bcl-2, with a low proliferation index determined by Ki67 staining (Fig. 4b, lower left). The urothelium had a relatively high proliferation index determined by Ki67 staining (Fig. 4b, approximately 60%). Diffuse immunoreactions of GATA-3 (Fig. 4c) and p53 (Fig. 4d) were also detected in the urothelium with severe dysplasia.

## Discussion and conclusions

PTs are rare neoplasms first described by Johannes Müller in 1838 [6]. They were temporarily named cystosarcoma phyllodes while emphasizing their benign biological behavior. In 1982, the World Health Organization classification recommended the use of the term phyllodes tumors for such lesions, and classified them into three types (benign, borderline, and malignant) to avoid overtreatment [7].

With the highest incidence in the breast, PTs account for less than 1% of all breast tumors [8]. The incidence of prostate PT ranks second [2, 3]. There is no clear statistical data on the morbidity of prostate PT. However, less than 100 prostate PT cases have been reported [1, 2]. More commonly found in the breast and prostate, primary PT has rarely been reported at other sites such as seminal vesicles and the verumontanum [2, 3].

PT is extremely rare in the urinary tract. In 2007, Oliveira et al. reported the first case of bladder PT in an experimental female rat at necropsy [5]. In 2018, Tchrakian et al. encountered the first and unique PT case of human bladder PT [4]. This was a 54-year-old man who underwent a partial cystectomy because of multiple recurrences. Histopathological examination revealed a polypoid epithelial–stromal biphasic tumor confined to the mucosal layer in the bladder dome. Epithelial and subepithelial stromal cells showed neither noticeable atypia nor obvious mitotic figures. No heterologous components or infiltrative growth was observed. This bladder tumor was classified as low grade/benign PT.

To our knowledge, primary PT in the ureter has not been previously reported. In our case, the leaf-like pattern and epithelial–stromal biphasic differentiation closely resembled breast PT. The stromal cells in our case were stellate and spindle-shaped in appearance, showing a non-infiltrative border, bland cytology, rare mitotic figures, and a low proliferation index, all of which were in accordance with benign lesions.

In this case, what appeared more concerning was the urothelial epithelium with moderate-to-severe dysplasia and early infiltration. In breast PT, malignant transformation usually occurs in the stromal component and is less common in the epithelial component. Breast PT cases accompanied by carcinoma are mainly associated with malignant PT. However, ductal carcinoma in situ (DCIS) and invasive ductal carcinoma (IDC) can also rarely occur in benign PT [9, 10]. In our case, the fissures were focally lined by urothelial epithelium with severe dysplasia, active mitotic activity, abnormal giant cells, and early infiltration, which was in accordance with the diagnosis of invasive urothelial carcinoma (IUC).

We deliberated on whether to make a direct diagnosis of PT for this case. Considering the rarity of PT in the urinary tract and much rarer epithelial malignant transformation of PT, a diagnosis of ureteral PT should be approached with caution in this case. We are unaware of ​​the specific location of benign PT in the bladder [4]. However, in contrast to the endodermal origin of most bladder mucosae, the ureter and the mucosa of the bladder trigone are embryologically derived from the ureteric bud, which is a derivative of the mesonephric duct [11]. The mesonephric duct origin of these two sites, which is the same as the mesodermal origin of the prostate, seminal vesicles, and verumontanum, may be a rational explanation for PT arising from these sites. Therefore, although no definitive diagnosis was made in this case, we propose some supporting evidence for the possibility of PT in the ureter and bladder.

Histological grading of PT predicts the short-term prognosis, while the overall recurrence rate of prostate PT is significantly higher than that of breast PT [12, 13]. Locally aggressiveness, sarcomatous changes, and distant metastases are also found in PT patients [13]. Therapeutically, PT is primarily treated by surgical resection in the breast, which can be extensive breast-conserving therapy or a total mastectomy [12]. Adjuvant radiotherapy effectively reduces local recurrence in patients with malignant PT, tumors of > 5 cm, age of < 45 years, tight margins, and breast preservation. The effects of chemotherapy in PT remain uncertain [14]. If DCIS or IDC is present in a breast PT, management shifts from wide local excision to further staging work-up including sentinel lymph node biopsy and radiation therapy [10].

Because our case is the first ureteral tumor with the morphological characteristics of breast PT, there are no established criteria for a treatment approach. Our patient underwent nephroureterectomy and partial cystectomy and was free of symptoms at 7 months after surgery. Considering the potential for local recurrence of PT and IUC, long-term clinical and radiological follow-up of such lesions is advisable.

## Data Availability

Not applicable.

## Abbreviations

PT: Phyllodes tumor
CTU: Computed tomography-urography
IUC: Invasive urothelial cancer
DCIS: Ductal carcinoma in situ
IDC: Invasive ductal carcinoma

## References

[1] Bannowsky A, Probst A, Dunker H, Loch T (2009). Rare and challenging tumor entity: phyllodes tumor of the prostate. J Oncol.

[2] Tang J, He L, Long Z, Wei J (2015). Phyllodes tumor of the verumontanum: a case report. Diagn Pathol.

[3] Xu LW, Wu HY, Yu YL, Zhang ZG, Li GH (2010). Large phyllodes tumour of the seminal vesicle: case report and literature review. J Int Med Res.

[4] Tchrakian N, Browne E, Shanks JH, Flynn RJ, Crowther S (2018). Phyllodes tumour of the urinary bladder: a report of a unique case. Histopathology.

[5] Oliveira PA, Colaco AA, Palmeira CA, De la Cruz PL, Lopes CA (2007). A phyllodes tumor of the urinary bladder in a rat. Exp Oncol.

[6] Fiks A. Cystosarcoma phyllodes of the mammary gland--Müller's tumor. For the 180th birthday of Johannes Müller. Virchows Arch A Pathol Anat Histol. 1981;392(1):1-6. 10.1007/BF00430543 6269275

[7] Histological typing of breast tumors (1982). Tumori.

[8] Parker SJ, Harries SA (2001). Phyllodes tumours. Postgrad Med J.

[9] Lui SA, Oh HB, Wang S, Chan CW (2018). Ductal carcinoma in-situ arising within benign phyllodes tumours. Ann R Coll Surg Engl.

[10] Panko N, Jebran AA, Gomberawalla A, Connolly M (2017). Invasive Ductal Carcinoma within a Benign Phyllodes Tumor. Am J Case Rep.

[11] Jeffus S (2014). Histology for Pathologists, 4th Edition. Am J Surg Pathol.

[12] Mishra SP, Tiwary SK, Mishra M, Khanna AK (2013). Phyllodes tumor of breast: a review article. ISRN Surg.

[13] Bostwick DG, Hossain D, Qian J, Neumann RM, Yang P, Young RH, di Sant’agnese PA, Jones EC (2004). Phyllodes tumor of the prostate: long-term followup study of 23 cases. J Urol.

[14] Bogach J, Shakeel S, Wright FC, Hong NJL (2022). Phyllodes tumors: a scoping review of the literature. Ann Surg Oncol.

